# Modular structure of brain functional networks: breaking the resolution limit by Surprise

**DOI:** 10.1038/srep19250

**Published:** 2016-01-14

**Authors:** Carlo Nicolini, Angelo Bifone

**Affiliations:** 1 University of Verona, Verona, Italy; 2 Istituto Italiano di Tecnologia, Center for Neuroscience and Cognitive Systems, Rovereto (TN), Italy

## Abstract

The modular organization of brain networks has been widely investigated using graph theoretical approaches. Recently, it has been demonstrated that graph partitioning methods based on the maximization of global fitness functions, like Newman’s Modularity, suffer from a resolution limit, as they fail to detect modules that are smaller than a scale determined by the size of the entire network. Here we explore the effects of this limitation on the study of brain connectivity networks. We demonstrate that the resolution limit prevents detection of important details of the brain modular structure, thus hampering the ability to appreciate differences between networks and to assess the topological roles of nodes. We show that Surprise, a recently proposed fitness function based on probability theory, does not suffer from these limitations. Surprise maximization in brain co-activation and functional connectivity resting state networks reveals the presence of a rich structure of heterogeneously distributed modules, and differences in networks’ partitions that are undetectable by resolution-limited methods. Moreover, Surprise leads to a more accurate identification of the network’s connector hubs, the elements that integrate the brain modules into a cohesive structure.

The brain is often represented as a network of interconnected, dynamically interacting elements^1^. Cognitive processes are thought to result from the integration of neuronal processing distributed across these complex networks at different temporal and spatial scales^2^. Hence, comprehension of the organizational principles of brain networks may provide a key to understand the interplay between functional segregation and integration, and ultimately the emergence of cognition and adaptive behaviors.

Neuroimaging methods provide a powerful means to study the brain structural and functional architecture. Indeed, neuroimaging data can be naturally represented as networks, or graphs, with image voxels or anatomically defined regions corresponding to the nodes, and a measure of similarity or connectedness between nodes representing the edges. By way of example, correlations between spatially remote changes in the BOLD signal measured by Magnetic Resonance Imaging have been used to define the strength of functional connectivity between different brain regions^3^. Similarly, white matter fibers interconnecting different brain regions can be traced by Diffusion Tensor Imaging to build the brain structural connectivity network^4^. The application of graph theoretical methods to the analysis of neuroimaging data has provided important insights into the topological organization of the central nervous system, and is attracting increasing attention as a general and powerful framework to analyze brain connectivity networks^1^.

Of particular interest is the study of the modular structure of brain networks, i.e. the presence of subsets, or clusters, of nodes that are more densely connected among themselves than to nodes in other modules^5^. This concept originated in the study of social relationships and is sometimes referred to as “community detection”^6^. In the context of neuronal networks, communities can be interpreted as functionally or structurally segregated modules^7^,^8^, a feature that is thought to confer robustness and adaptivity to the overall brain network^5^.

Several methods have been proposed to resolve the community structure of complex networks^6^,^9^. Many of these methods involve the definition of a quality function that assigns positive or negative scores to edges connecting nodes within or outside the same community, and heuristics to find the optimal partition of the network that maximize this fitness function. The most popular approach is Newman’s “Modularity maximization” and variations thereof^10^. Following the first demonstration by^11^, partitioning of brain networks using Modularity has been widely applied to assess the brain modular structure. A few, large modules, including the Default Mode Network, the central network, occipital and frontoparietal networks have been observed with remarkable consistency across subjects and studies^5^,^12^.

Despite its popularity and merits, Newman’s approach presents some important limitations. Already at an early stage, Modularity-based methods were shown to suffer from a resolution limit, as they fail to identify modules that are smaller than a scale that depends on the size of the overall network^13^. As a consequence, even unambiguously defined modules, like complete sub-graphs or cliques, may be unduly merged into larger communities when they are too small compared to the size of the network. Subsequent work by various groups has shown that the resolution limit is quite pervasive^9^,^14^,^15^,^16^,^17^, and affects, to a different extent, many other methods, including Reichardt and Bornholdt’s^18^, Arenas and Gomez’^19^, Ronhovde and Nussinov’s^20^, Rosvall and Bergstrom’s (*Infomap*)^17^,^21^ and others.

Fixes have been proposed to circumvent the resolution limit, including the introduction of a tunable parameter that enables analysis of the network at an adjustable resolution level^18^,^22^,^23^. However, this requires prior knowledge of the expected size of the communities for the tuning of the resolution parameter. Moreover, it has been shown that an adjustable resolution parameter may reduce the tendency to merge small clusters, but only at the cost of unduly splitting large clusters^16^. Adjustment of the resolution parameter is an attempt to balance these two biases, but multiresolution methods fail to recover community structures comprising heterogeneous distributions of cluster sizes^16^.

However, real-world networks are characterized by the coexistence of clusters of very different sizes, and no single parameter can adapt to the variety of network topologies observed in nature. Hence, the resolution limit may represent a critical shortcoming for the study of brain networks and is likely to have affected many of the studies reported in the literature.

Here, we explore the use of Surprise, a recently proposed fitness measure grounded in probability theory, for the study of brain functional networks. Surprise has been shown to outperform other metrics in the detection of small communities^24^,^25^,^26^, but the extent to which it is affected by the resolution limit is unclear. We show that, for graphs of the typical kind and size encountered in the study of brain connectivity, Surprise does not suffer from the limitations of Newman’s Modularity, and behaves as a resolution-limit-free fitness function.

Application of Surprise maximization to the partition of diverse brain connectivity networks reveals rich modular structures that comprise modules of heterogeneous sizes, including large, distributed clusters, and functionally segregated clusters of nodes that are very small compared to the size of the graph. We discuss the important implications of these findings for the identification of brain structures responsible for the integration of brain connectivity, and argue that current models of the brain modular architecture based on graph theoretical approaches may have suffered from the shortcomings of the resolution limit and should be revisited.

## Theory

### Notation and definitions

Let *G* = (*V*, *E*) be an unweighted, undirected graph, with *n* nodes and *m* edges and *p* pairs of vertices. A clustering *ζ* of *G* is a partitioning of *V* into disjoint sets of vertices 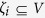 which we call communities. Each community consists of *n*_*i*_ vertices, *m*_*i*_ edges and *p*_*i*_ pairs of vertices. The number of total intracluster edges *m*_*ζ*_ and intracluster pairs *p*_*ζ*_ are respectively the sum of *m*_*i*_ and *p*_*i*_ over all communities.

If we take a graph 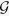 drawn uniformly at random from all possible graphs with the same vertex set *V* and exactly *m* edges, the probability that 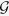 has at least *m*_*ζ*_ intracluster edges and *p*_*ζ*_ intracluster pairs is given by the inverse cumulative hypergeometric distribution:


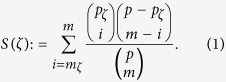


Equation 1 corresponds to an urn model without reinsertion, where *S* is the probability of extracting at least *m*_*ζ*_ white balls out of *m* trials from an urn containing *p*_*ζ*_ white balls and *p* − *p*_*ζ*_ black balls. For a clustering *ζ*, the function *S*, from Surprise, computes the probability to observe in an uniform random graph at least as many internal edges and pairs as in 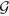. Intuitively, the lower *S*(*ζ*), the better the clustering. It’s worth noting that *S* is the *p*-value of a Fisher exact-test assessing how confidently one should reject the null hypothesis that the intracluster density *m*_*ζ*_/*p*_*ζ*_ is the same as the graph density *m*/*p*. Optimal partitions with respect to *S* are those with the highest number of intracluster edges and the smallest number of intracluster pairs. Due to numerical precision problems in the evaluation of large binomial coefficients, 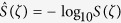 is often taken as measure of quality of the partition, with higher values corresponding to better clustering. Different authors^27^,^28^, refer to *S* as Surprise, whereas others^24^,^25^ use 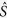. Hereafter we stick to the notation of 24, where Surprise is indicated as 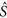. Hence, in this notation, the optimal partition of a graph is the one that maximizes 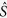.

### The resolution limit and Surprise

**Figure 1:**
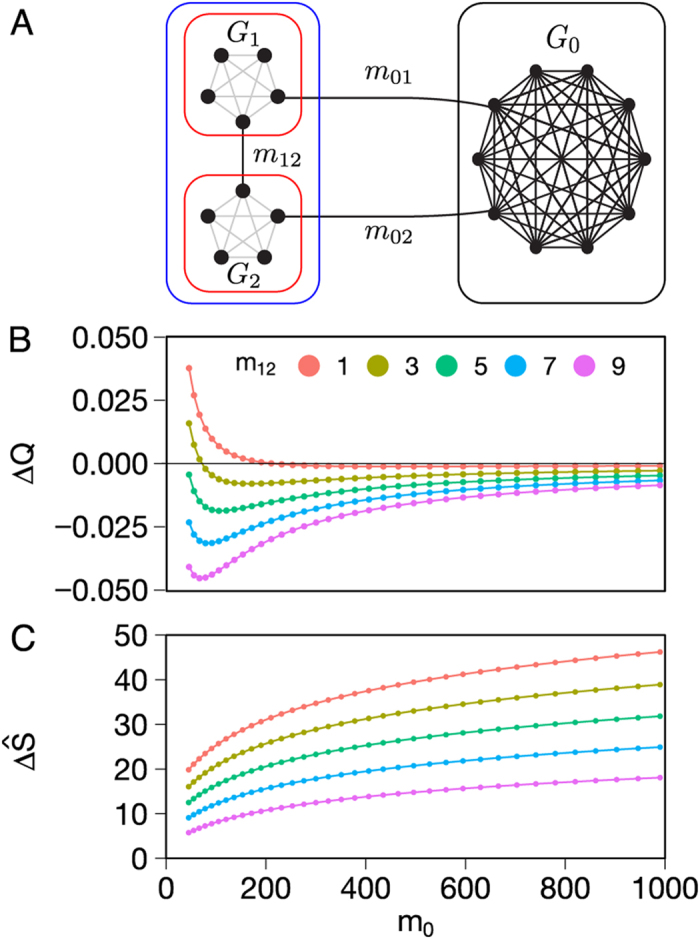
Analysis of the onset of the resolution limit for Modularity and Surprise in a model graph (**A**) consisting of two cliques, *G*_1_ and *G*_2_, and a size-varying components *G*_0_. The red line indicates the partition *α*, with *G*_1_ and *G*_2_ as different modules, and the blue line the partition *β*, with *G*_1_ and *G*_2_ merged into a single module. The graph (**B**) shows the difference in Modularity for increasing number of edges in *G*_0_. The same is shown in (**C**) for Surprise.

Fortunato and Barthelemy^13^ first detected the resolution limit studying the performance of Newman’s Modularity as a community detection method applied to a graph *G* with *m* edges consisting of three subgraphs *G*_0_, *G*_1_, *G*_2_ where *G*_*i*_ = (*V*_*i*_, *E*_*i*_), with |*V*(*G*_*i*_)| = *n*_*i*_ and |*E*(*G*_*i*_)| = *m*_*i*_ (Fig. 1A). The connections between the components are represented by *m*_01_, *m*_02_, *m*_12_, respectively. While *G*_1_ and *G*_2_ are modules by construction, *G*_0_ may consist of many communities.

To illustrate the resolution limit, Fortunato and Barthelemy calculated the values of Modularity *Q* in two different cases: in partition *α*, *G*_1_ and *G*_2_ were considered as distinct communities, while in partition *β* they were merged into the same module; the partition of *G*_0_ was arbitrary and identical in both cases. As *G*_1_ and *G*_2_ are two different modules by construction, *Q*_*α*_ is expected to be larger than *Q*_*β*_ in all cases. However, it was shown that *Q*_*β*_ can exceed *Q*_*α*_ when the number of internal edges *m*_1_ and *m*_2_ is small compared with the total number of edges in the graph *m*, thus preventing detection of small communities even when they are complete graphs or cliques. Subsequently, other authors have extended this analysis showing that the resolution limit affects a number of other community detection algorithms, and suggesting that the problem may be quite generally related with the use of non-local fitness functions^9^,^14^,^15^,^16^.

The resolution limit first highlighted by Fortunato and Barthelemy may be particularly critical for the analysis of brain connectivity networks. By way of example, certain functional processes, like color vision, have been described as anatomically localized^29^, while others, like working memory, have been proposed to involve more globally integrated processing systems^30^. Hence, we may expect the brain modular structure to comprise heterogeneously distributed communities.

Whether the relatively uniform modular structure of brain connectivity, highlighted by Newman’s Modularity and other community detection methods in many studies, reflects the true architecture of the brain organization or is the result of the resolution limit is still unclear. Hierarchical approaches have shown that large modules can be further subdivided, indicating that connectivity networks show structure at different spatial scales^31^. However, these findings do not provide information on the optimal partition of the network, i.e. the optimal cut through the dendrogram representing connectivity at the different scales. To this end, an optimization method that does not suffer from the resolution limit would be needed.

Unfortunately, the resolution limit appears to be an intrinsic feature of many methods that optimize global quality functions, and there appears to be “a narrow scope to resolution-limit-free methods”^14^. Surprise has been shown to outperform other network partitioning methods in the detection of small features within large graphs, but the extent to which it suffers from the resolution limit is unknown^24^,^25^,^26^. As pointed out by^24^, while Modularity-based methods define a community as a region with an unexpectedly high density of links with respect to the global characteristics of the network, Surprise weights the number of actual intracluster edges against the maximum number of links given the nodes in the clusters. Hence, Surprise is able to discriminate local subnetworks whose internal density is close to that of a clique independently of their size. In the following, we assess the extent to which the resolution limit may affect Surprise.

Firstly, we have directly compared Newman’s Modularity and Surprise in the example of Fortunato and Barthelemy. For the sake of illustration, we have defined *G*_1_ and *G*_2_ as two identical cliques of 5 nodes connected to *G*_0_ by a single edge (*m*_01_ = 1, *m*_02_ = 1), and to each other by *m*_12_ edges. *G*_0_ was defined as a clique of variable size with a number of edges ranging from 45 to 2775. We then computed the numerical difference of the quality functions Modularity and Surprise for the two partitions *α* (red) and *β* (blue) as in Fig. 1A and plotted Δ*Q* = *Q*_*α*_ − *Q*_*β*_ and Δ*S* = *S*_*α*_ − *S*_*β*_ as a function of the number of edges *m*_0_ in the *G*_0_ clique. These plots are shown in Fig. 1B,C.

The onset of the resolution limit occurs when Δ*Q* or Δ*S* change sign and become negative for increasing values of *m*_0_. For *m*_12_ = 1, i.e. when the two cliques *G*_1_ and *G*_2_ were connected by only one edge (red curve), *Q* showed this sign inversion for *m*_0_ ≈ 200 (Fig. 1B). With increasing number of intercluster edges *m*_12_, the resolution limit appeared for smaller values of *m*_0_, eventually leading to Δ*Q* values that were always negative, i.e. the two cliques *G*_1_ and *G*_2_ were always merged by Modularity optimization.

Figure 1C shows that Surprise does not suffer from the resolution limit in this specific case. Indeed, Δ*S* was always positive and grew monotonically with increasing *m*_0_. Hence, the two cliques *G*_1_ and *G*_2_ were always resolved by Surprise as separate communities independently of the network size, and also in the presence of some “fuzziness”, i.e. when *m*_12_ > 1 and the two cliques were connected by more than one edge. In order to assess whether this behavior reflects a general property of Surprise, or is incidental to this particular example, we have also studied a generalization of Fortunato and Barthelemy’s model.

Traag *et al.*^14^ proposed a rigorous definition of resolution-limit-free graph partitioning. A quality function is resolution-limit-free if, given an optimal partition *ζ* of a graph *G*, any subpartition *ζ*_*i*_ is also optimal for the graph induced by the nodes in *ζ*_*i*_. In other words, each community of the optimal partition is not split by optimization of the quality function applied to the subgraph induced by the nodes in the community. Hence, each community does not depend on the rest of the network and is both locally and globally optimal.

**Figure 2:**
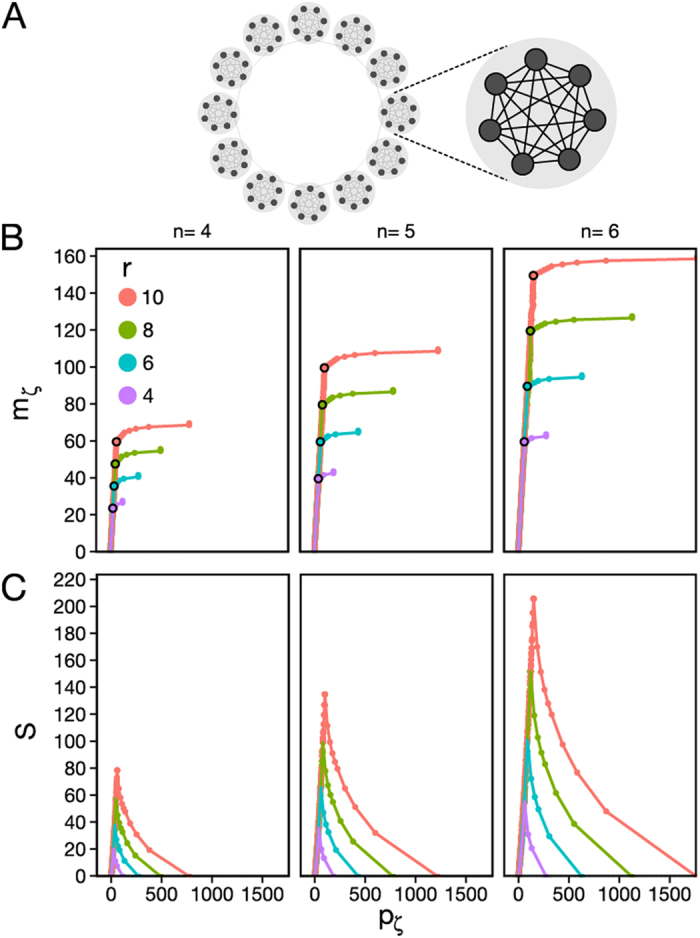
Behavior of Surprise for different partitions of a ring of cliques (**A**) of varying size. *n* denotes the number of nodes in each clique, and *r* the number of cliques in the graph. (**B**) shows the Pareto frontier for various values of *n* and *r*. The black circle corresponds to the optimal partition by Surprise. (**C**) shows the value of Surprise for each point of the Pareto frontier. The peak value corresponds to the optimal partition where each clique of the ring represents a separate module.

An important consequence of this definition is that a resolution-limit-free method will never depend on the size of the network to merge cliques in a graph comprising *r* cliques of *n* nodes connected in a ring structure as in Fig. 2A.

This observation prompted us to explore the behavior of 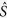 in the ring of cliques model graph, as an extension of Fortunato and Barthelemy’s model. Surprise optimization can be seen as a multiobjective optimization problem where one seeks to minimize the intracluster pairs while maximizing the number of intracluster edges. With increasing graph size, the computational problem of calculating 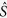 for every possible partition becomes rapidly intractable (maximization of *S* is *NP* hard)^28^. However, as pointed out by Fleck *et al.*^28^, the *S*-optimal clustering must be Pareto optimal with respect to minimizing *p*_*ζ*_ and maximizing *m*_*ζ*_, i.e. any further improvement in one of the two variables must occur at the expense of the other.

To delineate the Pareto frontier in the (*m*_*ζ*_, *p*_*ζ*_) space for the ring of cliques, we solved *m* integer linear programs where we sought to minimize *p*_*ζ*_ while keeping *m*_*ζ*_ equal to a constant *k*, with *k* ranging from 0 (trivial partition where every vertex is a community) to *m* (trivial partition with all vertices in the same community). Linear programs were solved using the Python interfaces of Gurobi 5.7.3 on Linux (Gurobi Optimizer Version 5.7, Gurobi Optimization, Inc., Houston, Texas, United States).

Figure 2B shows the Pareto frontier for a ring of cliques where we independently varied the number of cliques *r* and the number of nodes *n* in every clique. Interestingly, 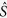 increased monotonically along the Pareto frontier with increasing *p*_*ζ*_ (Fig. 2C), until it reached its optimum, indicated by black circles in the Pareto frontier, for the partition that identified each clique as a separate community. Importantly, in the range of parameters we have investigated, Surprise optimization never merged cliques in the ring of cliques, independently of the size of the graph, and behaved as a Traag’s resolution-limit free method. While it is likely that this property is quite general and can be extended to every ring of cliques, an analytical demonstration is hampered by the non-additivity of the Surprise function. Nonetheless, the size of the graphs we have explored numerically is quite typical of brain-connectivity networks and we feel encouraged to apply Surprise maximization to the study of the community structure of the brain.

## Methods

### Surprise maximization

Community detection is a NP-hard problem, and heuristics have to be developed for the optimization of quality functions for relatively large networks. In their original paper, Aldecoa *et al.*^24^ applied metaheuristics, involving the evaluation of S for partitions resulting from seven different community detection methods, each of those maximizing different quality functions. Here, we sought direct maximization of Surprise by exploiting FAGSO^32^, an agglomerative optimization algorithm that builds on a variation of the Kruskal algorithm for minimum spanning tree^33^. The first step of this method consists in ranking the edges in the graph in decreasing order by the Jaccard index of the neighbors of their two endpoints vertices. An union-find data structure is used to hold the community structure throughout the computation. At the beginning, each community consists only of one vertex. Then, starting from the edge with the highest Jaccard index at the top of the list, the endpoints are attributed to the same community by disjoint-set union if this operation leads to a strictly better Surprise and if they do not belong already in the same community. This step is repeated for all edges and the final community structure is returned in the disjoint-set. This method finds partitions with high Surprise and it is deterministic, unless two edges with the same Jaccard index are found. In this case, ties are broken at random. The detailed pseudocode of this algorithm is reported in the Supplementary Materials section, the code in C++, Python and GNU Octave is available upon request.

### Benchmark brain networks

We assessed the performance of Surprise maximization in the detection of the community structure of two benchmark brain networks. All coordinate data and functional metadata were taken from the BrainMap database^34^,^35^, processed by Crossley *et al.*^36^ and made available to the scientific community as reference networks through the public Brain Connectivity Toolbox^37^. Ethical statements are present in the original references by the groups who performed the experiments.

The first network represents the coactivation of brain regions as obtained from a meta-analysis of 1641 task-related fMRI or PET studies^36^. Meta-analyses have been useful in estimating the frequency with which two brain regions are consistently activated across different tasks and are an indication of the behavior of the brain during activity. Jaccard similarity, i.e. the number of studies activating both regions divided by the number of studies activating either one of them, was used as index to evaluate strength of the coactivation of 638 parcellated brain regions. More details on the construction of the network are available in^36^.

The second network that we considered is a resting state functional connectivity network obtained from correlations between time series of fMRI signals, from a group study of 27 healthy subjects. The resting state network was built using the same set of 638 regions and thresholded to have the same number of edges as in the coactivation study. Both networks have been previously studied using Modularity-based algorithms and node-classification methods^36^.

Due to its definition in terms of binomial coefficients, Surprise can be computed for integer values of its parameters. We have therefore binarized the two adjacency matrices retaining an equal number of edges for both networks. While the binarization process discards information contained in the edge weights, a judicious choice of threshold can ensure robust decomposition of the network^5^,^38^. We have checked this statement by percolation analysis, a natural and non-arbitrary method to derive binary graphs from continuous adjacency matrices. Specifically, we have studied the size of the largest connected component of the coactivation and resting state networks removing iteratively the smallest weight edges.

This analysis, shown in Figure S1 of the Supplementary Materials, revealed the presence of percolation-like transitions, whereby the largest component of the network drops in jumps with increasing binarization threshold. For the coactivation and the resting state networks we found that the thresholds adopted by^36^ of 0.015576 and 0.600, respectively, are above the first jump in the size of the largest connected components and maintain network connectedness while ensuring that the networks are sufficiently sparse and possess the same number of edges. Hence, we adopted these thresholds for network’s binarization. Analysis of the structures of networks obtained by a range of thresholds around these values showed stable solutions, with Normalized Mutual Information close between partitions close to 1, and a stable number of communities (Figures S2 and S3 in the Supplementary Information).

## Results and Discussions

**Figure 3:**
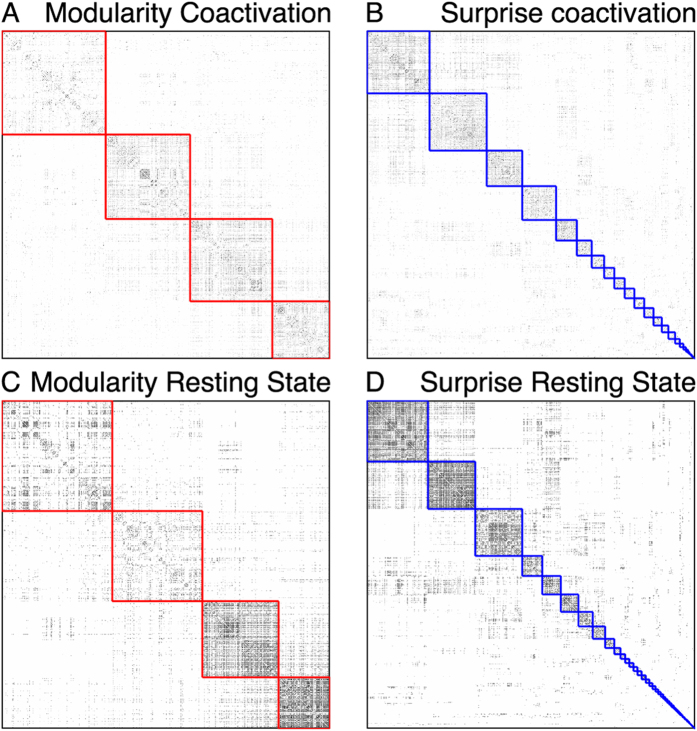
Modular structure of the coactivation and resting state networks under Modularity and Surprise maximization. The node indexes have been reordered by membership to highlight the modules, which are demarcated by a red line, for Modularity, or a blue line, for Surprise. Modularity maximization identifies only four, large modules, consistent with previous analysis of these data-sets. Surprise reveals a much finer and complex modular structure.

Figure 3 shows a direct comparison of the partitions obtained by Modularity and by Surprise maximization for the coactivation and resting state networks. The four panels display the adjacency matrices of the two networks, with their vertices rearranged by their module membership.

By Newman’s decomposition, the resting state and coactivation brain networks present a modular structure with four large modules that have been anatomically labeled as occipital, central, frontoparietal and Default Mode networks^36^ (demarcated by a red line in Fig. 3). These partitions are highly similar (Rand Index 0.78), despite the different neurofunctional bases of the two networks^39^ and comprise modules that are relatively uniform in terms of number of nodes and number of edges within each module.

The partitions obtained by Surprise maximization for the two networks are shown in Fig. 3B,D. Surprise found 51 communities, 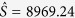, for the resting state network, and 28 communities, 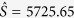 for the coactivation network. These modules are delimited by blue lines that show the wide distribution in size of the components, ranging from communities with 119 nodes and 4586 edges down to singletons. The size distributions of the modules are different for the two networks, with a more rapid drop and a fatter tail in the coactivation network compared with the resting state network.

The complete list of communities, with anatomical labels and stereotaxic coordinates for all nodes^40^,^41^,^42^, as well as the density and number of nodes of each community found by Modularity and Surprise optimization, are reported in a tabular form in the Supplementary Materials (Tables S1–S4).

Analysis by Surprise suggests that the modular structure of resting state functional connectivity brain networks comprises modules of very different sizes, in sharp contrast with previous studies that have used resolution-limited functions like Newman’s Modularity (see^5^ for a review). To emphasize this point, we have also partitioned the coactivation and resting state networks using Infomap^21^ and a multiscale version of Modularity with an adjustable resolution parameter^18^ provided by the Brain Connectivity Toolbox^37^. Interestingly, increasing the resolution parameter results in a larger number of smaller communities that are however characterized by a relatively homogenous size distribution, a result of the intrinsic scale built into these methods (results shown in the Supplementary Material, Figure S4). Additionally, we have made a quantitative comparison between the partions obtained by Surprise, Infomap and the Reichardt and Bornholdt’s method^18^ by calculating the Normalized Mutual Information between the resulting community structures (Tables S6 and S7 in the Supplementary Material). Despite the fact that these methods retrieve a few more modules that Newman Modularity, they fail to capture the heterogeneous distribution of clusters revealed by Surprise.

**Figure 4:**
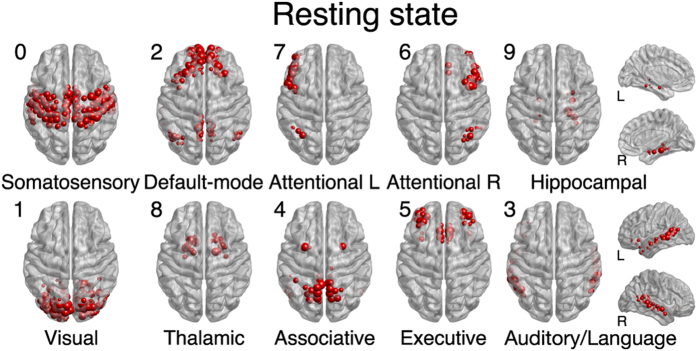
The ten largest modules found by Surprise in the resting state network overlaid on an MRI brain template. The module indexes are ordered by decreasing size. The modules are named after corresponding functional networks previously identified by multivariate analysis of resting state fMRI data.

In order to assess the significance in neurofunctional terms of the finer partitions obtained by Surprise, we show the node distribution as an overlay of the MNI brain atlas template for the 10 largest modules of the resting state network in Fig. 4. The communities highlighted by Surprise show a correspondence with some well known functional networks previously identified by multivariate analysis (e.g. Independent Component Analysis) of functional MRI data^43^,^44^,^45^,^46^, and with well defined, segregated anatomical or functional districts.

The largest communities of the resting state network correspond to the primary sensorimotor cortex^47^, primary visual and extra-striate visual network, fronto-parietal lateralized networks^39^ as well as the so-called default mode network (DMN)^43^,^48^. The attentional frontoparietal networks (FPAN)^49^ were detected as two separate, lateralized subnetworks, in agreement with^46^ although other studies have identified a single, bilateral FPAN^50^.

Smaller networks, like the executive control and auditory networks^44^,^51^ were also resolved by Surprise, as well as subcortical structures, like the hippocampal and thalamic formations^52^,^53^. Interestingly, the thalamic nuclei appear as one tight community, despite the fact that they are structurally unconnected, in keeping with the idea that functional connectivity does not necessarily require the presence of strong structural links.

**Figure 5:**
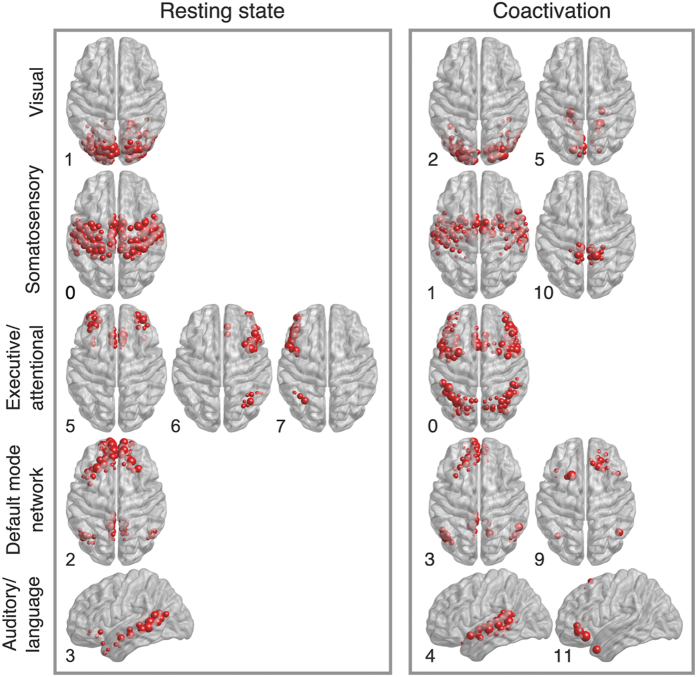
Comparison of selected modules in the partition obtained by Surprise in the resting state and coactivation networks. The indexes are inversely ranked according to the size of the modules in their respective networks.

The more accurate partition afforded by Surprise may enable identification of differences in the modular structures of networks that cannot be appreciated with a resolution limited method. By way of example, we have compared the partitions of the resting state and coactivation networks (Fig. 5). Indeed, these networks are of a different nature, the former representing intrasubject baseline fluctuations in the brain’s resting state, and the latter the responses to a variety of different tasks across subjects. However, Newman’s Modularity finds similar partitions for these two networks, with 4 large modules each. Conversely, under Surprise maximization, the partition of the resting state network shows many more small communities comprising less than 5 nodes (32 in total) compared with the coactivation one (only 11). Moreover, certain communities of the resting state network appeared to be split into smaller modules in the coactivation matrix. By way of example, the cuneus and the lingual and pericalcarine gyri were part of the occipital visual module in the resting state, but not in the coactivation network, where they formed a separate community (first row of Fig. 5). Similarly, the precuneus and medial parts of the postcentral girus were identified as an independent community in the coactivation network, while they were part of the broad somatosensory network in the resting state connectivity graph^54^ (second row of Fig. 5). Interestingly, the Broca area, indicated as Module 11 in Fig. 5, was separated from the auditory network in the coactivation network, and identified as a small, but anatomically and functionally distinct, community. Conversely, other communities were split in the resting state but not in the coactivation network. The executive and attentional control networks were merged into a large community in the coactivation network, while they were separated under resting state conditions, including a subdivision of the left and right fronto-parietal networks (third row of Fig. 5).

While the resting state and coactivation networks appeared to possess virtually identical modular structures under Newman’s analysis, they showed functionally and anatomically relevant differences when analyzed by Surprise maximization, with a Normalized Mutual Information between the partitions of the two networks of 0.5922. Indeed, Modularity tends to assign small communities to larger structures even when they correspond to tightly knit modules, thus concealing differences in the graphs’ modular structures that involve aggregation or disaggregation of smaller clusters. It is conceivable that the detrimental effects arising from the resolution limit may have affected previous studies comparing different populations^5^. Surprise may offer a sharper tool to detect alterations of brain connectivity induced, for example, by psychiatric or neurological conditions, thus enabling the exploration of novel markers of brain disease.

Besides the exploration of functional and anatomical segregation, understanding the modular structure of brain networks is critical for the interpretation and classification of the roles played by the nodes within the network structure^55^. Highly connected nodes, or hub nodes, are particularly important for their topological centrality, and function as integrators. Hubs that primarily connect to nodes within the same module are dubbed “provincial hubs”, and nodes that connect different modules are referred to as “connector hubs”. The former are thought to be responsible for the formation and stability of the modules, while the latter ensure integration of the activity of the different modules. Obviously, interpretation of the hub’s role relies on the correct identification of the optimal network partition, and may be strongly affected by the resolution limit.

Here, we have performed a hub’s role analysis for the resting state and coactivation networks under Modularity and Surprise maximization. To this end, we have adopted Guimera’ and Amaral classification scheme^56^, whereby nodes are classified by their within-community degree *z* (a measure of how well connected a node is to other nodes in the same community) and their participation coefficient *P*, a parameter that is 0 for nodes with purely intra-module connections and 1 for nodes whose links project primarily to other modules.

**Figure 6:**
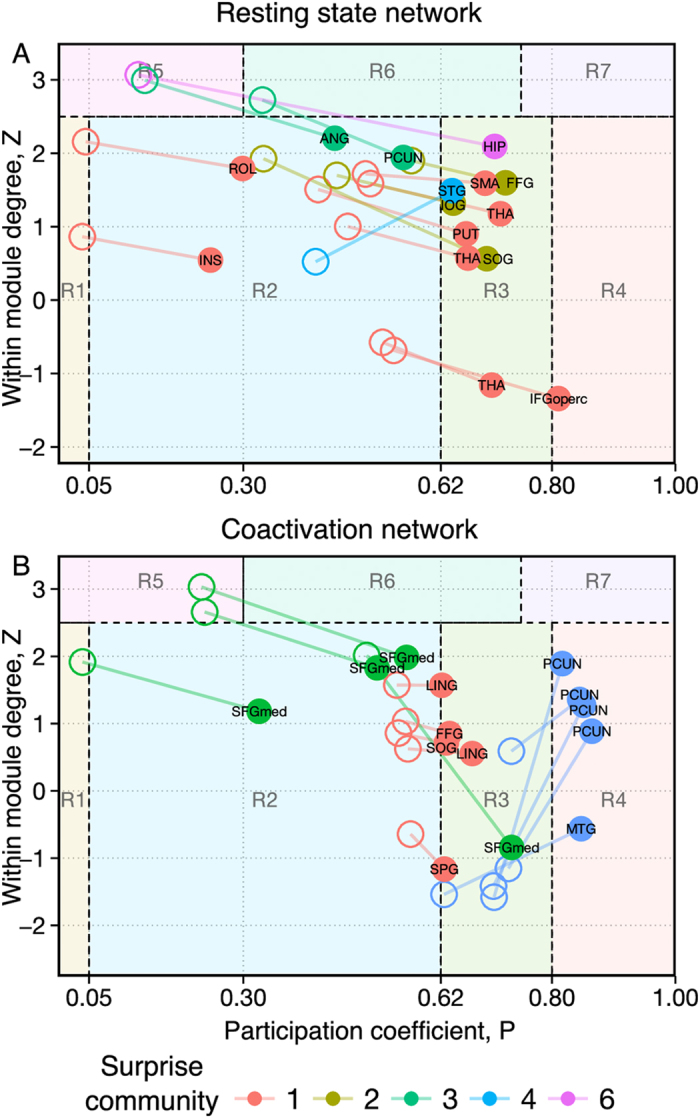
Classification of representative nodes according to their intra- and intermodule connections for the resting state (**A**) and coactivation (**B**) networks. Empty circles and full circles indicate the position of each node in the Guimera’ and Amaral’s plot after partition by Modularity or Surprise, respectively. An overall increase in the participation coefficient, a measure of the intermodule connectivity, is observed for the Surprise partition relative to the Modularity partition. To avoid cluttering of the graph, we only reported those nodes with a degree higher than the average within a Standard Deviation, and whose classification is different in the two partitions. The abbreviations of the brain regions corresponding to the nodes are reported in the Supplementary material, Table S5.

Figure 6A,B show the different positioning of high-degree nodes in the Guimera’ and Amaral plot for the coactivation and resting state networks partitioned using Newman’s approach and Surprise maximization. In this scheme, provincial hubs are high-degree nodes that score high *z* and low *P* values (*R5* region); conversely, connector hubs are characterized by larger *P* values (regions on the right-hand side of the plot).

A finer partition in smaller communities may be expected to determine an overall increase in participation coefficient and decrease in within-community degree. However, the heterogeneous partitions obtaind by Surprise maximization resulted in non-trivial changes in the Guimera’ and Amaral classification. By way of example, we discuss in greater detail two regions whose roles are very different in the two partitions, to highlight the effects of the resolution limit.

Nodes that belong in the hippocampal formation show a large within-module coefficient, and appear as provincial hubs (region R5 of Fig. 6) under Modularity optimization. However, their participation coefficient increases 6-fold in the partition by Surprise, which reveals a role as connectors for these nodes, with widespread projections to many other modules across the brain, including the module 3, 6, 8, 5, 2, corresponding to the DMN, the amygdala and parahippocampal formation, the temporal inferior gyrus, the cuneus and lingual gyrus, and the visual cortex, respectively. This finding is in agreement with the idea that the hippocampus acts as “network convergence zone”, as it receives polysensory input from distributed association areas in the neocortex^57^.

**Figure 7:**
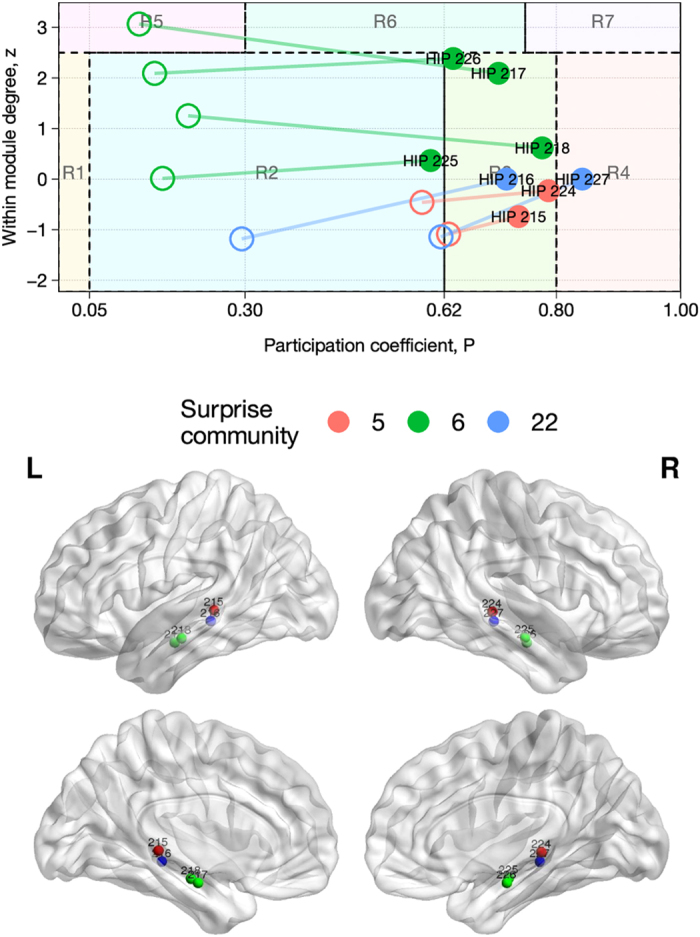
Top panel: classification of all the hippocampal nodes according to the Guimera’ and Amaral’s scheme for the coactivation network. Empty circles and full circles indicate the position of each node after partition by Modularity or Surprise, respectively. Bottom panel: anatomical positions of the nodes in the hippocampal formation, colored by Surprise community membership. The increase in participation coefficient upon partition by Surprise is more pronounced for nodes in the anterior part of hippocampus, with an antero-posterior gradient.

Interestingly, the right shift in the Guimera-Amaral plot is less pronounced for the nodes in the posterior part of the hippocampus (Fig. 7). A differential classification of the anterior and posterior hippocampus is consistent with the hypothesis of a functional differentiation of this structure^58^, with the posterior hippocampus mostly involved in memory and cognition, and the anterior hippocampus playing a role in the processing of stress, emotion and affect^59^. Moreover, studies in animal models have shown differential organization of the efferent connections of the hippocampal formation^60^, consistent with different functions for the anterior and posterior hippocampus.

Similar rightward shifts for nodes and hubs were observed in the resting state network, and reported in Fig. 6. However, increases in participation coefficients are by no means the only differences in the classification of nodes obtained by Surprise maximization. A prominent example is the precuneus (PC) (Fig. 6B, blue dots) that shows a high participation coefficient in both partitions by Modularity and Surprise, but a much higher within-module degree under Surprise maximization.

**Figure 8:**
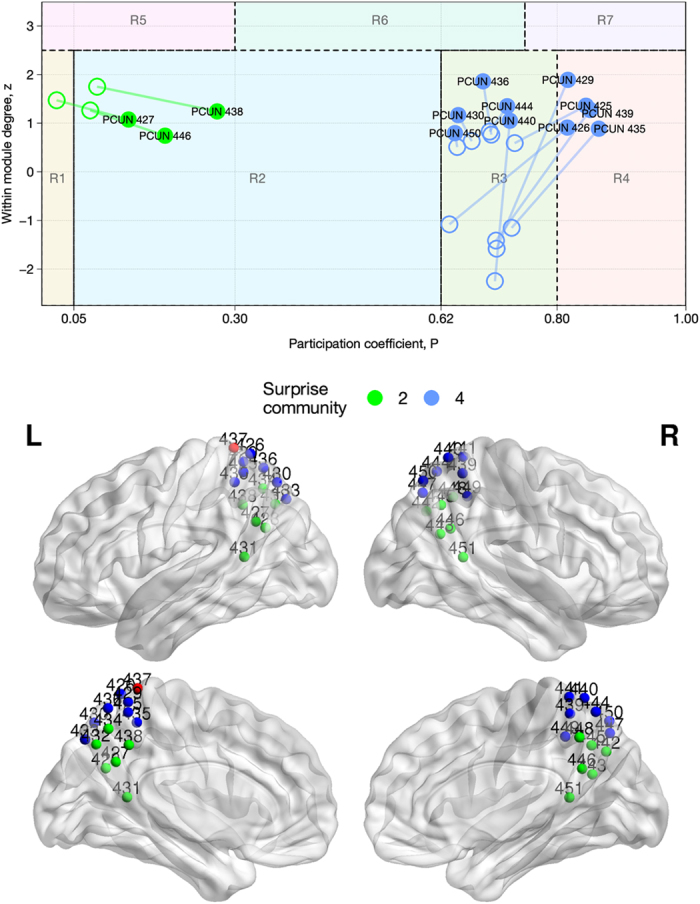
Top panel: classification of the precuneal nodes according to the Guimera’ and Amaral’s scheme for the resting state network. Empty circles and full circles indicate the position of each node after partition by Modularity or Surprise, respectively. Bottom panel: anatomical positions of the nodes in the precuneus, colored by Surprise community membership. The nodes in the dorsal part of the precuneus exhibit a sharp increase in within-module degree.

Indeed, the nodes comprised in the precuneus intrinsically possess high inter-cluster connectivity, but are distributed among the four modules found by Modularity. In the partition by Surprise they are grouped together, and this precuneal module as a whole plays a connector’s role integrating different communities (Fig. 8), a hypothesis that is consistent with the precuneus supporting a wide spectrum of highly integrated tasks, from visuo-spatial imagery to episodic memory retrieval and self-processing operations^61^.

In summary, partition by Surprise maximization results in very different distributions of participation coefficients and within-module degree compared to Modularity. These differences are not uniform across nodes, and arise from the limited ability of Modularity to identify small modules. Finer partition by Surprise reveals very different roles for some key brain areas, and suggests that a systematic reanalysis of the topological roles of brain nodes and hubs may be in order.

### Limitations

A potential limitation of Surprise is related to its definition in terms of discrete probability distributions. This makes Surprise suited for the study of binarized networks. While the topological backbones of the networks we have investigated appear quite robust against removal of lowest-weight edges and binarization, as shown by our percolation and stability analyses (Supplementary Information, Figures S1, S2, and S3), an extension to weighted networks would be desirable. A recent observation^26^ on the relation between Surprise and the relative entropy between two probability distributions suggests that an asymptotical expansion of Surprise may overcome this limitation, and enable application of Surprise maximization to weighted networks as well.

The superior resolution afforded by Surprise may make it more vulnerable than other methods to noise and experimental errors. Indeed, occasional mis-assignments of nodes due to noise-induced changes in edge distribution are likely to affect small modules, comprising only a few nodes, more than large ones. Hence, experimental uncertainty also limits resolution, and a resolution-limit-free method would not necessarily improve the quality of the partition in a scenario dominated by noise.

To ascertain whether this is the case for the co-activation and resting state networks investigated here, we have simulated the effects of experimental errors by injecting noise into the distributions of weights prior to the binarization procedure, thus introducing variability in the connectivity structure of the resulting binary networks. We set levels of noise sufficient to perturb up to 10% of the edges of the final binary network. Using this procedure, for each level of noise we generated ten different graphs, and applied the Surprise Maximization algorithm to each of them. We found that the partitions of these graphs were highly consistent with those of the original networks (Fig. S5 in the Supplementary Information). We should also stress that there is no constraint in the FAGSO algorithm imposing inter-hemispherical symmetry of the partition. Nevertheless, we observed homotopic correspondence in the community structure, and a close resemblance with established neurofunctional circuits (Figs 4 and 5). Taken together, simulations of the effects of noise and qualitative considerations on the neurofunctional significance of the modules identified by Surprise corroborate the idea that experimental error is not the predominant factor in the networks investigated in this paper.

A final and important point we should highlight is that Surprise maximization, in the implementation we have used here, does not allow for overlapping communities. Other methods have been applied to investigate this aspect in brain networks^62^,^63^. However, a recent comparative analysis of graph partitioning algorithms on a variety of benchmark networks^9^ has shown that these methods are also prone to merging overlapping communities, with relatively modest performance in recovering heterogeneous cluster distributions planted in model networks.

Despite these potential limitations, the resolution-limit-free behavior of Surprise makes it an excellent tool to explore and to overcome the effects of the resolution limit in the modular structure of brain connectivity networks.

## Conclusions

In conclusion, we have shown that Surprise, a recently proposed fitness function for graph partitioning, behaves like a resolution-limit-free function. We have applied Surprise maximization to study the modular structures of two different brain networks. Surprise maximization resulted in partitions comprising communities of widely distributed sizes, consistent with the idea that small and large modules coexist in brain networks. Moreover, the finer partition afforded by Surprise made it possible to appreciate differences in the modular structures of diverse brain networks that were undetected by resolution limited methods like Newman’s Modularity. Finally, the use of Surprise revealed the deleterious effects of the resolution limit in the classification of nodal roles. Altogether, these results indicate that the resolution limit may have substantially affected many of the analyses of brain connectivity networks reported in the literature, and call for a revisitation of some of the conclusions and models that have relied on Modularity maximization or similarly resolution-limited algorithms. Surprise appears as a promising alternative method that appeals to the intuition that tightly-knit clusters of nodes represent legitimate structural or functional modules independently of their size.

## Additional Information

**How to cite this article**: Nicolini, C. and Bifone, A. Modular structure of brain functional networks: breaking the resolution limit by Surprise. *Sci. Rep.* **6**, 19250; doi: 10.1038/srep19250 (2016).

## Supplementary Material

Supplementary Information
