# Supplementary material for: Modular structure of brain functional networks: breaking the resolution limit by Surprise
Source: Sci Rep. 2016 Jan 14;6:19250. doi: 10.1038/srep19250 (PMC4725862; doi:10.1038/srep19250)
Supplement: Supplementary Information [file srep19250-s1.pdf]

Modular structure of brain functional networks: breaking the resolution limit by Surprise

Carlo Nicolini, Angelo Bifone

Supplementary Materials

Table S1: Modularity Coactivation

Community	Density	$\lvert E_c \rvert$	$\lvert V_c \rvert$
0	0.122457	2486	202
1	0.311761	4167	164
2	0.321541	4090	160
3	0.342825	2131	112

Table S2: Modularity Resting state

Community	Density	$\lvert E_c \rvert$	$\lvert V_c \rvert$
0	0.154097	3545	215
1	0.109951	1674	175
2	0.489336	5323	148
3	0.671313	3323	100

Table S3: Surprise Coactivation

Community	Density	$\|E_c\|$	$\|V_c\|$
0	0.491261	3626	122
1	0.482883	2948	111
2	0.553282	1298	69
3	0.388811	834	66
4	0.573077	447	40
5	0.458128	186	29
6	0.442029	122	24
7	0.394737	75	20
8	0.452632	86	20
9	0.397661	68	19
10	0.415205	71	19
11	0.379085	58	18
12	0.541667	65	16

Table S4: Surprise Resting State

Community	Density	$\|E_c\|$	$\|V_c\|$
0	0.653183	4586	119
1	0.755375	3162	92
2	0.543101	2224	91
3	0.495277	367	39
4	0.560317	353	36
5	0.616756	346	34
6	0.513228	194	28
7	0.525362	145	24
8	0.601307	92	18
9	0.454545	30	12
10	0.583333	21	9
11	0.678571	19	8
12	0.607143	17	8

Table S5: Abbreviation of brain regions.

Region	Abbreviation
ACG	Anterior Cingulate Gyrus
CAL	Calcarine
CAU	Caudate
CUN	Cuneus
DCG	Median cingulate and paracingulate gyri
FFG	Fusiform Gyrs
HES	Heschl Gyrus
HIP	Hippocampus
IFGoperc	Inferior Frontal Gyrus, opercular part
IFGtriang	Inferior Frontal Gyrus, triangular part
INS	Insula
IOG	Inferior Occipital Gyrus
IPL	Inferior Parietal Gyrus
ITG	Inferior Temporal Gyrus
LING	Lingual Gyrus
MFG	Middle Frontal Gyrus
MOG	Middle Occipital Gyrus
MTG	Middle Temporal Gyrus
ORBinf	Inferior Frontal Gyrus, orbital part
ORBmid	Middle Frontal Gyrus, orbital part
ORBsup	Superior Frontal Gyrus, orbital part
ORBsupmed	Superior Frontal Gyrus, medial orbital
PAL	Globus Pallidum
PCG	Posterior cingulate Gyrus
PCL	Paracentra Lobule
PCUN	Precuneus
PHG	Parahippocampal Gyrus
PoCG	Postcentral Gyrus
PreCG	Precentral Gyrus
PUT	Putamen
REC	Rectus
ROL	Rolandic Operculum
SFG	Superior Frontal Gyrus
SFGmed	Superior Frontal Gyrus, medial part
SMA	Supplementary Motor Area
SMG	Supramarginal Gyrus
SOG	Superior Occipital Gyrus
SPG	Superior Parietal Gyrus
STG	Superior Temporal Gyrus
THA	Thalamus
TPOmid	Temporal Pole, Middle temporal Gyrus
TPOsup	Temporal Pole, Superior temporal Gyrus

Algorithm 1: Pseudocode of the Surprise maximization algorithm.

```
FAGSO(G)
S ← 0 ▷ Initialize Surprise to 0
D ← ∅ ▷ Initialize disjoint set forest
for each vertex v in V[G]
        do MAKE-SET(v)
E' ← SORT-JACCARD(E) ▷ Sort edges in decreasing order by Jaccard index
for each edge(u, v) ∈ E', ▷ Taken in decreasing order by Jaccard index
        do if FIND-SET(u) ≠ FIND-SET(v)
                then if SURPRISE(G, D ∪ {(u, v)}) > S
                        D ← D ∪ {(u, v)}
                        UNION(u,v) ▷ Merge the communities u and v belong
                        S = SURPRISE(G, D) ▷ Update current Surprise
return D

MAKE-SET(x)
p[x] ← x
rank[x] ← 0

LINK(x, y)
if rank[x] > rank[y]
    then p[y] ← x
    else  p[x] ← y
            if rank[x] = rank[y]
                then rank[y] ← rank[y] + 1

UNION(x, y)
LINK(FIND-SET(x),FIND-SET(y))

FIND-SET(x)
if x ≠ p[x]
    then p[x] ← FIND-SET(p[x])
return p[x]

SURPRISE(G, D)
m_ξ ← 0 ▷ Number of intracluster edges
p_ξ ← 0 ▷ Number of intracluster pairs of vertices
m ← |E[G]| ▷ Number of edges
p ← binom(|V[G]|, 2) ▷ Number of pairs of vertices
for each g in CONNECTED-COMPONENTS-SUBGRAPHS(D, G)
        do m_ξ ← m_ξ + |E[g]|
            p_ξ ← p_ξ + binom(|V[g]|, 2)
return −log_10 ( sum_{i=m_ζ}^{m} binom(p_ξ, i) binom(p − p_ξ, m − i) / binom(p, m) )
```

Line 8 of SURPRISE:

$$\textbf{return } -\log_{10}\left(\sum_{i=m_\zeta}^{m}\frac{\binom{p_\xi}{i}\binom{p-p_\xi}{m-i}}{\binom{p}{m}}\right)$$

Table S6: Normalized Mutual Information between partitions of the coactivation network with Newman's Modularity, Infomap, Reichardt and Bornholdt and Surprise. We used the Fagso algorithm for Surprise maximization, the `igraph` implementation of Infomap, the Brain Connectivity Toolbox implementation for RB (`community_louvain.m` function) and `modularity_und.m` for Modularity. For each method the best solution over 100 repetitions was used to calculate NMI.

Coactivation

**NMI**	Modularity	Surprise	Infomap	RB $\gamma = 1.75$
Modularity	**1.00**	0.61	0.56	0.61
Surprise	0.61	**1.00**	0.54	0.62
Infomap	0.56	0.54	**1.00**	0.59
RB $\gamma = 1.75$	0.59	0.61	0.59	**1.00**

Table S7: Normalized Mutual Information between partitions of the Resting state network with Newman's Modularity, Infomap, Reichardt and Bornholdt and Surprise. We used the Fagso algorithm for Surprise maximization, the `igraph` implementation of Infomap, the Brain Connectivity Toolbox implementation for RB (`community_louvain.m` function) and `modularity_und.m` for Modularity. For each method the best solution over 100 repetitions was used to calculate NMI.

Resting State

**NMI**	Modularity	Surprise	Infomap	RB $\gamma = 1.75$
Modularity	**1.00**	0.52	0.70	0.66
Surprise	0.52	**1.00**	0.53	0.58
Infomap	0.70	0.53	**1.00**	0.68
RB $\gamma = 1.75$	0.66	0.58	0.68	**1.00**

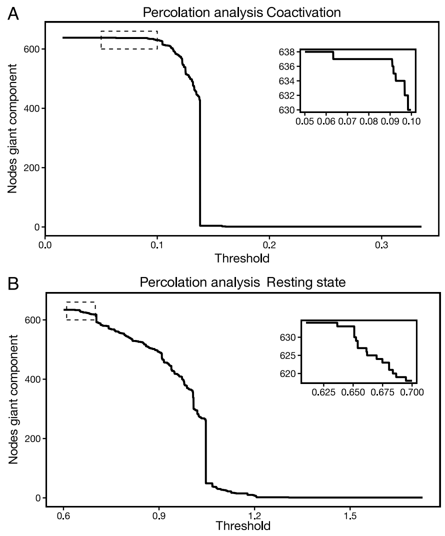

Figure S1: Percolation analysis for the coactivation matrix (A) and resting state matrix (B).

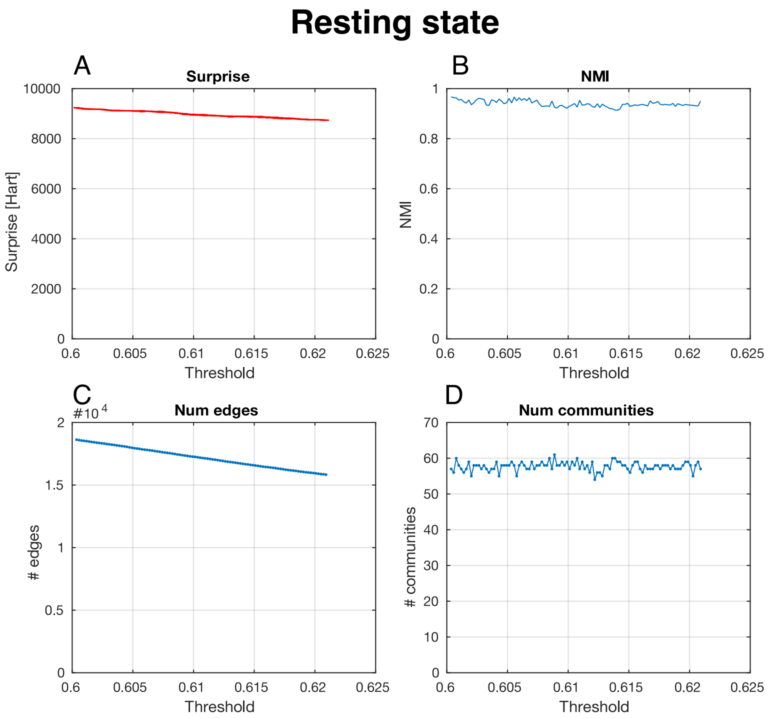

Figure S2: Different Resting State networks were generated varying the binarization threshold in a range that changed the number of edges up to the 15th quantile of the edge weight distribution. This corresponds to threshold ranges of 0.015-0.03 for the co-activation network, and 0.60-0.62 for the resting state network. The resulting networks were partitioned by Susrprise maximization. The value of Surprise, the Normalized Mutual Information between the resulting optimal partitions, the number of edges and the number of communities are reported in panel A, B, C and D, respectively.

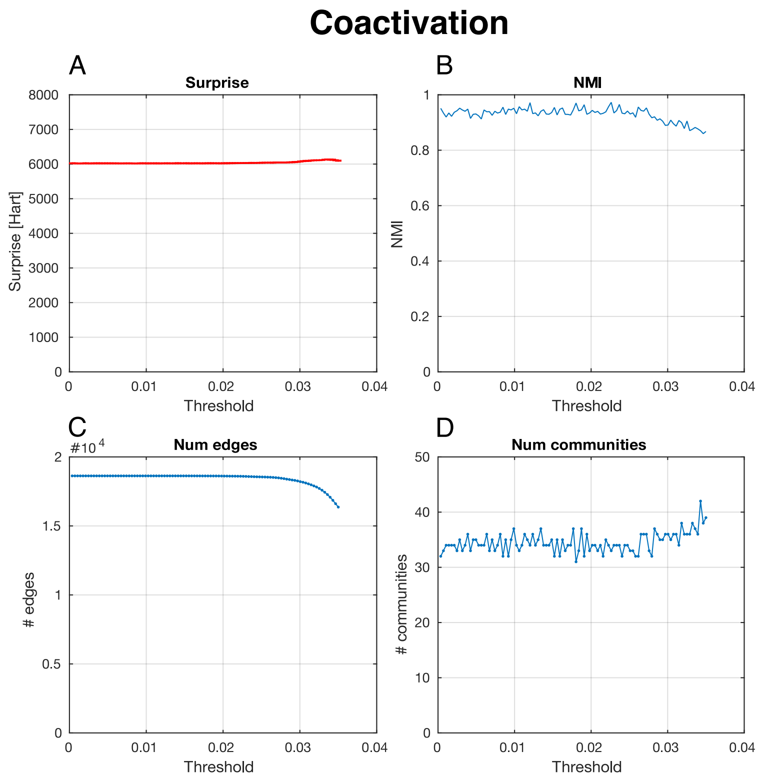

Figure S3: Different coactivation networks were generated varying the binarization threshold in a range that changed the number of edges up to the 15th quantile of the edge weight distribution. This corresponds to threshold ranges of 0.015-0.03 for the co-activation network, and 0.60-62 for the resting state network. The resulting networks were partitioned by Susrprise maximization. The value of Surprise, the Normalized Mutual Information between the resulting optimal partitions, the number of edges and the number of communities are reported in panel A, B, C and D, respectively.

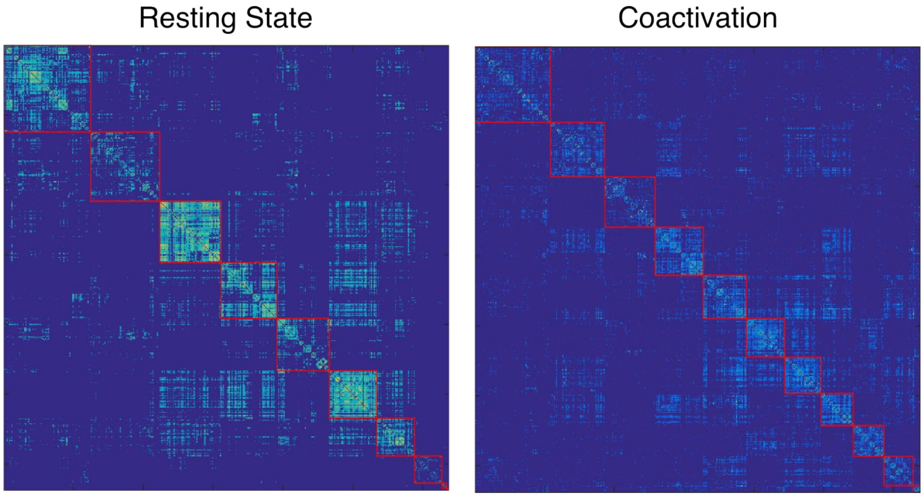

Figure S4: Partitions obtained with multiresolution Modularity (Reichardt and Bornholdt method) for a value of the resolution parameter $\gamma = 1.75$ for the coactivation and resting state networks. Increasing the resolution parameter improves detection of smaller modules, but breaks up larger ones, thus resulting in relatively homogenous size distributions.

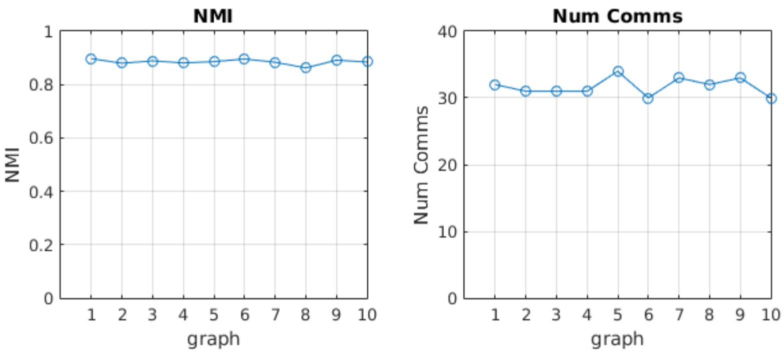

Figure S5: Assessment of the effects of experimental error on the community structure of the resting state network. Ten different graphs were generated by adding noise to the off-diagonal elements of the adjacency matrix prior to the binarization procedure. The amplitude of noise was chosen to randomly perturb 10% of the edges after binarization. Surprise maximization was applied to the resulting graphs. In the left panel, we report the Normalized Mutual Information (NMI) between the optimal partitions of each of the ten perturbed networks and that of the original resting state network. The number of communities retrieved by FAGSO in each of the perturbed networks is reported in the right panel. The graphs show that the partitions of the "noisy" graphs are consistent and similar to those of the unperturbed network, with NMI scores close to 1 and almost constant numbers of communities. These results demonstrate that Surprise maximization can retrieve the network's community structure even in the presence of substantial noise in the data.
